# Optimising Alzheimer's Disease Diagnosis and Treatment: Assessing Cost-Utility of Integrating Blood Biomarkers in Clinical Practice for Disease-Modifying Treatment

**DOI:** 10.14283/jpad.2024.67

**Published:** 2024-04-10

**Authors:** Sandar Aye, R. Handels, B. Winblad, L. Jönsson

**Affiliations:** 1 Division of Neurogeriatrics, Department of Neurobiology, Care Sciences and Society, Karolinska Institutet, BioClinicum J9:20, Akademiska stråket, 171 64, Solna, Sweden; 2 Department of Psychiatry and Neuropsychology, Maastricht University; Alzheimer Centre Limburg; Faculty of Health, Medicine and Life Sciences; School for Mental Health and Neuroscience, 6200 MD, Maastricht, The Netherlands; 3 Theme Inflammation and Aging, Karolinska University Hospital, 141 86, Stockholm, Sweden

**Keywords:** Cost-utility, blood biomarker, disease-modifying treatment, Alzheimer's disease

## Abstract

**Background:**

Recent developments in blood biomarkers (BBM) have shown promising results in diagnosing amyloid pathology in Alzheimer's Disease (AD). However, information on how these BBMs can best be used in clinical settings to optimise clinical decision-making and long-term health outcomes for individuals with AD is still lacking.

**Objectives:**

We aim to assess the potential value of BBM in AD diagnosis within the context of disease-modifying treatment (DMT).

**Design:**

We developed a decision analytic model to evaluate the long-term health outcomes using BBM in AD diagnosis. We compared standard of care (SOC) diagnosis workflow to the integration of BBM as a (1) referral decision tool in primary health center (PHC) and (2) triaging tool for invasive CSF examination in specialist memory clinic (MC). We combined a decision tree and a Markov model to simulate the patient's diagnostic journey, treatment decisions following diagnosis and long-term health outcomes. Input parameters for the model were identified from published literature and registry data analysis. We conducted a cost-utility analysis from the societal perspective using a one-year cycle length and a 30-year (lifetime) horizon.

**Measurements:**

We reported the simulated outcomes in the percentage of correct diagnosis, costs (in 2022 Euros), quality-adjusted life year (QALY), and incremental cost-effectiveness ratios (ICER) associated with each diagnosis strategy.

**Results:**

Compared to SOC, integrating BBM in PHC increased patient referrals by 8% and true positive AD diagnoses by 10.4%. The lifetime costs for individuals diagnosed with AD were € 249,685 and €250,287, and QALYs were 9.5 and 9.52 in SOC and PHC pathways, respectively. The cost increments were €603, and QALYs gained were 0.01, resulting in an ICER of €48,296. Using BBM in MC reduced the exposure to invasive CSF procedures and costs but also reduced true positive AD diagnoses and QALYs.

**Conclusions:**

Using BBM at PHC to make referral decisions might increase initial diagnostic costs but can prevent high costs associated with disease progression, providing a cost-effective DMT is available, whereas using BBM in MC could reduce the initial evaluation cost but incur high costs associated with disease progression.

## Introduction

**T**he approval of two new disease-modifying treatments (DMTs), aducanumab and lecanemab, by the US FDA (1, 2) and the potential approval of lecanemab by European Medicines Agency highlights the urgency to establish an accurate and cost-effective diagnostic pathway to identify eligible candidates for treatment. Since the drugs were tested in mild cognitive impairment (MCI) and mild dementia with amyloid abnormality (3, 4), early detection of these individuals is crucial to delay disease onset and reduce progression. This implies the necessity for screening and evaluation of a large population of MCI and mild dementia. The prevalence of this group is large, and health systems seem unprepared for screening this population for treatment (5, 6).

Diagnostic evaluation of Alzheimer's disease (AD) is typically initiated in a primary health centre (PHC) with referrals to specialist memory clinics (MC) (7). However, there is no standardised diagnostic pathway (8), and criteria for referral to MC are different across health systems. The National Institute for Health and Care Excellence (NICE) suggests referral of all suspected dementia after excluding the reversible causes for cognitive decline (9), while the Swedish guideline recommends referral only when the definitive cause is unclear (10). Moreover, pathologic confirmation is not mandatory in routine care (11), with diagnosis of AD often relying on clinical criteria, leading to misdiagnosis of pathology rates of up to 25–30% (12). With effective DMT for amyloid abnormality, the current diagnosis approach may be inefficient (13, 14). Current approaches for amyloid confirmation include CSF biomarkers (15) and positron emission tomography (PET) scans (16). However, the widespread use of these methods is limited by feasibility and cost, especially with the anticipated increase in demand with DMT availability. Moreover, if DMT is to be prescribed only at the specialist clinic, the referral approach also presents challenges, with the NICE approach potentially overwhelming MCs while the Swedish approach limits treatment access. Therefore, an affordable, scalable, and user-friendly diagnostic test is needed to facilitate referrals by excluding individuals with normal amyloid while identifying those with abnormal amyloid early and accurately in the diagnostic pathway.

Recent advancements in blood biomarkers (BBM) have shown promise for identifying amyloid abnormality, with some achieving diagnostic accuracy of over 90% compared to CSF amyloid tests (17). Several reviews have suggested BBM for screening individuals with memory complaints, guiding physician referrals and investigations, or replacing CSF or PET (18∼^20^). However, the accuracy and validity of BBM must be further studied before its use in practice (21). Moreover, using BBM as a referral decision tool also depends on the referral goal, as described by examples from the NICE and Swedish health care systems. Currently, the goal of PHC is to optimise AD diagnosis and to use referrals when needed. With an available DMT, we believe the goal of PHC is to optimise referral to MC to prevent system overload and for MC to identify amyloid to optimise treatment decisions. The value of BBM in this situation is yet to be explored. While prospective clinical studies are ongoing, decision-analytic modelling using existing data can provide insights into the potential value of BBM in this aspect.

Economic evaluation of BBM in AD diagnosis is currently limited. Existing models are either focused on the prescription of symptomatic AD treatment (22) or the intermediate outcomes like number of correct AD diagnoses (23). To conduct a comprehensive economic evaluation of diagnostic intervention, it is important to consider both the direct benefits or side effects linked to diagnoses and the indirect impacts arising from downstream treatment decisions guided by test results (24, 25). The existing models did not provide adequate insights into these aspects. This study aims to assess the potential value of integrating BBM into the AD diagnostic workflow, particularly in the context of DMT. We compared standard of care (SOC) diagnostic workup with the integration of BBM (1) in PHC's diagnostic workflow to guide referral to specialist MC, and (2) in MC's diagnostic workflow as a triaging tool for invasive CSF or PET diagnostic procedures.

## Methods

A decision-analytic model was created using TreeAge Pro 2023 healthcare version to evaluate the lifetime costs and benefits of using BBM for AD diagnosis. The model combines a decision tree, representing the AD diagnostic journey in PHC and MC, and a Markov model simulating the long-term health outcomes following the diagnosis where an effective DMT is prescribed to AD in MCI and mild dementia with abnormal amyloid. The analysis was conducted from a societal perspective with a one-year cycle length and a 30-year time horizon, aligning with the lifetime course of AD. All costs were reported in 2022 euros (€). Both costs and benefits were discounted at an annual rate of 3% (26) The willingness-to-pay (WTP) threshold for a quality-adjusted life year (QALY) was set at €50,000 (∼ 580,000 SEK) according to the Swedish dental and pharmacological agency's (TLV) history of decision for medicine reimbursement (27).

The diagnostic part of the model structure was established after critical review among the authors. For the dementia disease progression a widely used structure was adopted. Internal consistency was determined by running extreme value analyses. Oneway sensitivity analysis was conducted to investigate the impact of parameter uncertainties on the model's outcomes. The selection of specific parameters for this analysis was based on the authors' judgment of their potential influence on model outputs, and the findings were visually represented using a tornado diagram. To assess the robustness of the model results, a probabilistic sensitivity analysis with 10,000 iterations was performed with beta probability distribution applying to prevalence, sensitivity, specificity, test probabilities, and utility values, and gamma distribution to costs. The values of the parameters used in the sensitivity analysis were detailed in Table 1.

**Table 1:** Input parameters

Parameter	Value	Low value	High value	Unit	Probability distribution	Reference
Patient population						
Starting age (years)	65				Fixed	
Population distribution (%)						(28), calculation
SCD	0.53				Fixed	
MCI	0.3	0.25	0.35	Mean (95% CI)	Beta	
Mild dementia	0.17	0.14	0.20	±20%	Beta	
Prevalence of amyloid positivity (%)						(29)
SCD	0.31				Fixed	
MCI	0.55	50.1	59.5	Mean (95% CI)	Beta	
Dementia	0.84	77.5	89	Mean (95% CI)	Beta	
Referral rate						
Proportion of MCI referred to memory clinic in standard of care	0.52	0.416	0.624	±20%	Beta	Calculation from Svedem
Proportion of BBM positive MCI referred to memory clinic	1				Fixed	Assumption
Proportion of dementia referred to memory clinic in standard of care	0.52	0.416	0.624	±20%	Beta	calculation from Svedem
Proportion of BBM positive dementia referred to memory clinic	1				Fixed	Assumption
Blood biomarker test accuracy (reference CSF)						(32)
Sensitivity	0.89	0.8	0.95	Mean (95% CI)	Beta	
Specificity	0.69	0.54	0.81	Mean (95% CI)	Beta	
CSF test accuracy (reference PET)						(31)
Sensitivity	0.91	0.84	0.96	Mean (95%CI)	Beta	
Specificity	0.89	0.84	0.94	Mean (95%CI)	Beta	
Transition probability						
SCD to MCI in amyloid positive	0.41				Fixed	(35)
MCI to mild in amyloid positive	0.22				Fixed	(35)
SCD to MCI in amyloid negative	0.1				Fixed	(35) (36), calculation
MCI to mild in amyloid negative	0.05				Fixed	(35) (36), calculation
Between dementia states					Fixed	Analysis from SveDem, see Table S3
Mortality						
Age-specific population mortality					Fixed	Statistics Sweden
Hazard ratios of death due to AD						
SCD and MCI	1				Fixed	Assume same mortality rate with general population
Very mild AD	1.82				Fixed	(41)
Mild, moderate, severe and institutionalization					Fixed	Analysis from SveDem, see Table S4 & S5
Treatment effectiveness						
DMT effect	0.27	0.22	0.324	+20%	Beta	(4)
Health utility						
Amyloid negative SCD	0.87	0.86	0.89	Mean (95%CI)	Beta	(47)
Amyloid positive SCD	0.86	0.83	0.89	Mean (95% CI)	Beta	(47)
Amyloid negative MCI	0.71	0.64	0.78	Mean (95%CI)	Beta	(47)
Amyloid positive MCI	0.81	0.77	0.85	Mean (95%CI)	Beta	(47)
Mild	0.74	0.69	0.79	Mean (SD)	Beta	(48)
Moderate	0.59	0.47	0.71	Mean (SD)	Beta	(48)
Severe	0.36	0.18	0.53	Mean (SD)	Beta	(48)
Cost for diagnosis (2022 euros)						
Primary care -SCD	959				Fixed	(25)
Primary care -MCI	1,896				Fixed	(25)
Primary care -dementia	1,896				Fixed	(25)
CSF examination including cost for memory clinic visit	1,940	1,552	2,328	± 20%	Gamma	(25)
Memory clinic visit	524	419	629	± 20%	Gamma	(25)
Blood biomarker	200	100	300		Gamma	Assumption
Disease-stage specific cost (2022 euros)						
SCD	9,180					Assuming same cost as MCI
MCI	9,180	7,344	11,016	±20%	Gamma	(43)
Mild	27,014	21,348	32,681	Mean (95%CI)	Gamma	(44)
Moderate	30,824					(44)
Severe	35,471					(44)
Mild-long term care	81,383					(44)
Moderate-long term care	66,849					(44)
Severe-long term care	66,977					(44)
Treatment cost (2022 euros)						
Annual DMT cost	5,000	4,000	6,000	±20%		Assumption


NOTE. Parameter values used in univariate and probabilistic sensitivity analysis were derived from mean and standard deviation (SD) if available or varied at a 20% lower and higher range if SD is unavailable from the literature. Abbreviation: SCD, subjective cognitive decline; MCI, mild cognitive impairment; AD, Alzheimer's Disease; CSF, cerebrospinal fluid; DMT, disease-modifying treatment.

### Model structure

**Figure 1:**
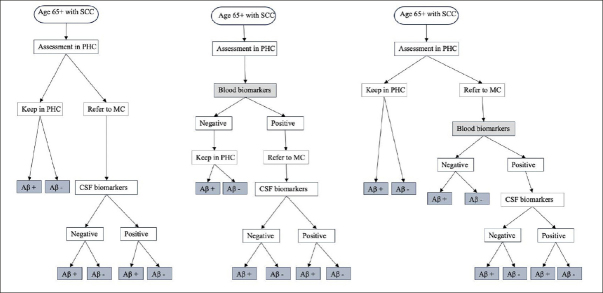
Conceptual framework for diagnosis journey of patients seeking care at primary health center NOTE. 1A. Standard of care pathway where patients are referred to MC based on PHC physician's decision. This was adapted from the Swedish clinical setting where only people with inconclusive basic dementia assessments, younger individuals, or those with unclear causes of mild cognitive impairment or dementia were referred to MC. 1B. Using blood biomarkers in PHC to guide clinician's decision for referral where people tested positive are referred to MC for further evaluation. 1C. Using blood biomarkers in MC to guide the decision for CSF examination where people referred to MC received BBM testing in addition to routine assessment by a specialist, only those who tested positive will continue with CSF examination. Abbreviation: SCC, subjective cognitive complaint; PHC, primary health centre; MC, memory clinic; Aβ, amyloid β; CSF, cerebral spinal fluid.

Figure 1 illustrates the conceptual framework for the patient journey. The population under study were people aged 65 years with subjective cognitive complaints (SCC) who underwent diagnostic evaluation in PHC and had not received a definitive AD diagnosis following the initial assessment. The interventions being evaluated were the use of BBM in PHC to guide clinicians' decisions for referral to MC and the use of BBM in MC as a triaging tool for CSF examination. The comparator was a simplification of the existing SOC diagnostic pathway in Sweden(10), described in detail in Section 1 in the supplementary file. In this SOC pathway (1A), individuals with SCC underwent standard diagnostic assessments in PHC without assessment of amyloid. Those judged inconclusive by the physicians regarding AD were referred to a MC where a definitive AD diagnosis was made based on CSF biomarker results. In the intervention pathway (1B), individuals with SCC underwent both standard diagnostic assessments as well as BBM in PHC. Those with positive BBM results were referred to a MC where a definitive AD diagnosis was made based on CSF biomarker results. In an alternative intervention pathway (1C), individuals with SCC underwent standard diagnostic assessments in PHC without assessment of amyloid. Those judged inconclusive by the physicians regarding AD were referred to a MC where BBM was used to guide the decision for CSF examination and only those with positive BBM results continued with CSF examination.

**Figure 2:**
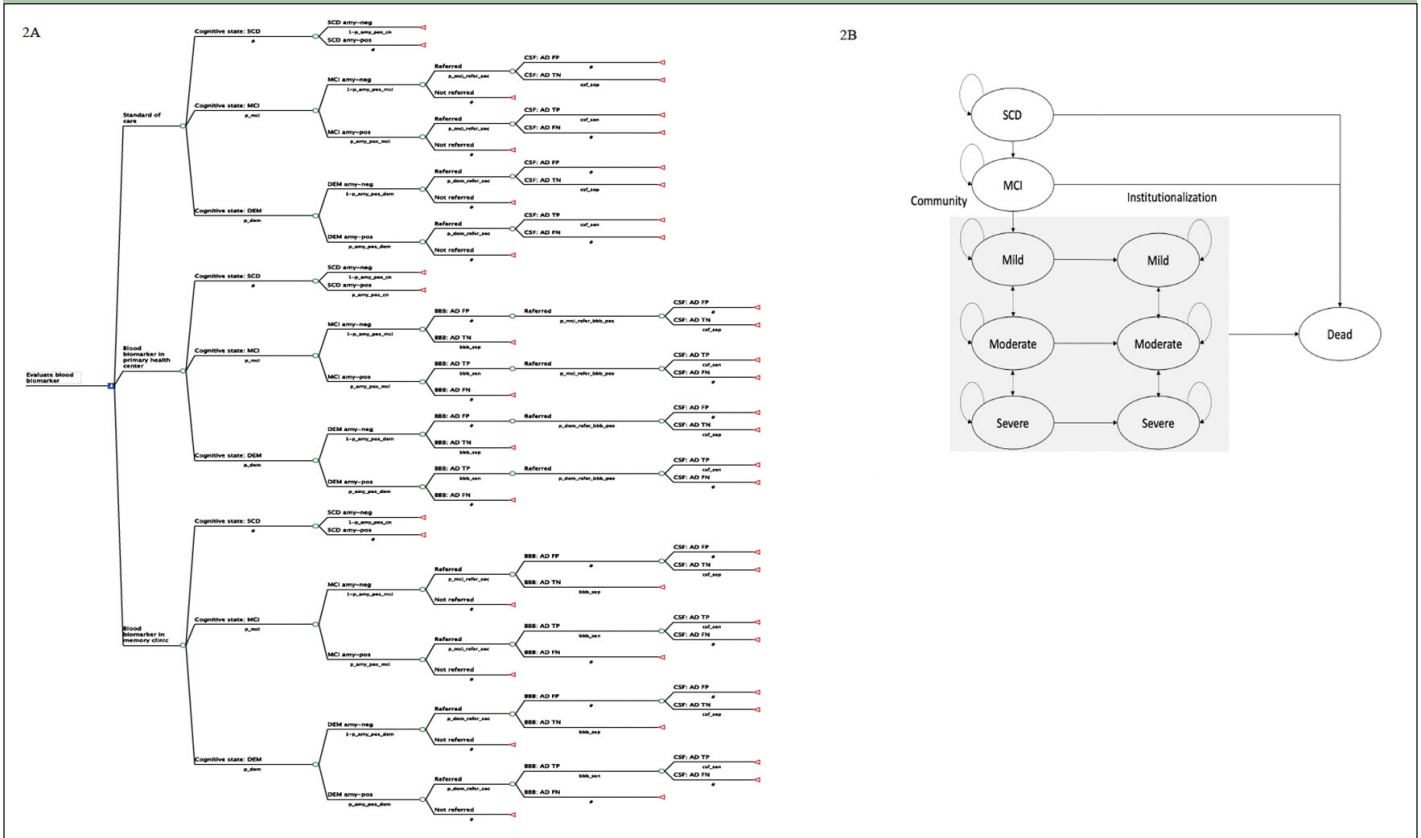
Model structure NOTE: 2A. Decision tree illustrating the diagnostic pathway in the standard of care and blood biomarker arms. 2B. State transition diagram with 9 health states used in the Markov model. Abbreviation: SCD, subjective cognitive decline; MCI, mild cognitive impairment; DEM, dementia; amy-neg, amyloid-negative; amy-pos, amyloid positive; BBM, blood biomarker; CSF, cerebrospinal fluid biomarker; AD, Alzheimer's disease; FP, false positive; TN, true negative; TP, true positive; FN, false negative.

Figure 2 depicts the model structure, including a decision tree and a Markov model, which are used to represent the diagnostic pathways and subsequent health outcomes for individuals seeking evaluation for SCC at PHC. Patients enter the decision tree at the age of 65 with SCC. The initial cognitive states (subjective cognitive decline (SCD), MCI, and Mild dementia) and amyloid statuses (amyloid-negative and amyloid-positive) were defined at the model entry using population prevalence (28) and amyloid positivity data (29). Individuals diagnosed with SCD (30) at PHC do not undergo further investigation due to the lack of available interventions at this clinical stage. In the SOC pathway, data from the Swedish dementia registry (SveDem) was used to model the probability of PHC physician referral decisions to MC. The final diagnosis of amyloid positivity relies on the sensitivity and specificity of CSF biomarker using PET as reference test (31) in MC. In the intervention pathways, the sensitivity and specificity of BBM using CSF as reference test (32) were used to model the probability of referral to MC or triage to CSF. Intermediate outcomes, such as costs for diagnostic evaluation, true positive (TP), false positive (FP), true negative (TN) and false negative (FN) which are direct results of each diagnostic strategy, were reported.

Individuals were categorised into ten groups based on their true health state, amyloid status and final diagnosis obtained from the decision tree. A summary of the final diagnoses and their subsequent clinical decision-making, costs and effects was presented in Table S1 in the supplementary file. A Markov model was then used to simulate the disease progression, treatment effects, costs and health outcomes. We used a 9-state Markov model (SCD, MCI, mild, moderate, severe, institutionalised mild, institutionalised moderate, institutionalised severe and dead) to represent disease progression, as shown in Figure 2B. In each annual cycle, they may remain in the same state, progress to a more severe state, regress to a less severe state or die based on the annual transition probabilities. Distinct annual progression rates were applied to amyloid-positive and amyloid-negative AD in the predementia states but not in the clinical dementia states. Transitioning to a less severe state was allowed in dementia states but not in MCI and mild AD as this reversion is more likely due to measurement errors rather than underlying neurodegeneration (33).

The following model assumptions were made.

The model considered only AD-type dementia. Individuals who tested amyloid-negative were categorised as ‘no AD' and were not subjected to any intervention. This patient group was considered to have stable MCI due to other causes and followed specific annual transition probabilities.

Treatment can only be provided after a biomarker-confirmed amyloid abnormality, which is not possible at PHC in the SOC pathway. This is because the goal of PHC in SOC is to refer only those judged as inconclusive. Hence, individuals clinically diagnosed with AD in PHC but not referred to MC were classified as FN diagnoses and did not receive DMT.

Amyloid positivity was considered the sole eligibility criterion for treatment, with no other exclusion criteria being considered. Therefore, 100% of people diagnosed with biomarker-confirmed AD received DMT.

Diagnosis was treated as a one-time process completed within one year, and individuals with incorrect diagnoses (both FP & FN) were not re-evaluated in the model.

CSF amyloid positivity was assumed independent (i.e., not correlated) to BBM amyloid positivity (e.g., in pathway 1B and 1C CSF sensitivity and specificity was the same for those BBM amyloid positive and BBM amyloid negative). Similarly, PHC inconclusiveness of AD diagnosis was assumed independent from true underlying amyloid status.

### Model parameters

Input parameters were derived from published literature and data analysis from SveDem as presented in Table 1.

#### Population characteristics

People aged 65 years who come to PHC for SCC do not have a definitive diagnosis of AD after initial evaluation with standard diagnostic procedures.

#### Disease prevalence

The prevalence estimates for MCI and dementia were derived from a Swedish population-based study (28). This study included a sample of 3,752 individuals aged between 60 and 99 years from the community. It provided detailed MCI prevalence and incidence data stratified into three age groups: 60, 70, and 80 years and older. For our model's population, we adopted the MCI prevalence value of 29.9% (95% confidence interval (CI): 25.1% - 34.8%) from the 70-year age group of the study. This study excluded individuals with dementia and those with impaired Instrumental Activities of Daily Living (IADL). To determine the prevalence of dementia, we calculated it based on the excluded population, resulting in a prevalence rate of 17%. It is worth acknowledging that the study represented a population sample and may not represent the population seeking care in PHC settings. However, another study conducted on individuals with SCD attending PHC in a Spanish population reported similar prevalence rates for MCI (28.2%) and dementia (13.3%) (34). Therefore, we made the assumption that these input parameters could reasonably reflect the situation in a Swedish PHC setting.

#### Amyloid positivity

The prevalence of amyloid positivity was derived from a meta-analysis of 85 study cohorts focused on amyloid biomarkers (29). This comprehensive study spanned both clinical and research cohorts and captured the full clinical spectrum of AD. The collective analysis involved 19,097 participants with an average age of 69 years. Each cohort employed specific CSF cutoff values to define amyloid abnormality. The pooled analysis provided data on amyloid abnormality prevalence, stratified by age groups and adjusted to cohort-specific CSF cutoffs. For individuals aged 65–70 years, the prevalence rates of amyloid positivity were 31.4% (95%CI: 24.4% – 39.3%) for those with SCD, 54.8% (95%CI: 50.1% – 59.5%) for those with MCI, and 84.1% (95%CI: 77.5% – 89%) for individuals diagnosed with clinical dementia.

#### Probability for referral

Regarding the probability of patient referral, we analysed data from SveDem and patient registry. We examined a cohort of 46,459 individuals registered in the primary health center module of SveDem between 2007 and 2022-03-02, representing individuals undergoing dementia evaluation in PHC. Subsequently, we cross-referenced these patients with the outpatient registry, which records clinic visits and diagnoses for administrative purposes, resulting in a total of 52,931 clinic visits. Utilising a two-by-two table approach, we found that 26.2% of individuals who initiated investigations in PHC had a documented diagnosis of any form of dementia in the outpatient registry. This figure represents the referral rate from PHC to MC for dementia diagnosis. In the context of an effective DMT, we assumed that the current practice referral rate would double equally in those with true underlying abnormal amyloid and normal amyloid, providing a 52% referral rate in the model.

#### Natural history of disease progression

##### SCD to MCI and MCI to dementia

We obtained annual conversion rates from amyloid-positive SCD and MCI to more severe states from a study by Potashman et al., which utilised data from the National Alzheimer's Coordinating Center (NACC) Uniform Data Set (35). The study defined health states using the Clinical Dementia Rating Sum of Boxes (CDR-SB) scale, categorising individuals into SCD, MCI, mild, moderate, and severe dementia. Transition probabilities between these health states were estimated for both incident and prevalent populations using multinomial logistic regression adjusted for factors like the time spent in each state, age, sex, and concurrent AD medications. In our model, we applied the estimates from the incident population, resulting in a 41% transition rate from SCD to MCI. For the transition from MCI to mild dementia, we combined all subcategories to simplify the model, resulting in a transition rate of 22%.

Annual transition probabilities for amyloid-negative SCD and MCI were derived from odds ratios (OR) for disease progression in amyloid-positive SCD (36) combined with the above transition probabilities. This meta-analysis involved eight studies that included SCD patients with at least one AD biomarker at baseline. The results demonstrated an increased risk of progression to MCI or dementia in the presence of amyloid pathology (OR: 5.89, 95%CI: 2.33–14.90). We converted these odds ratios to transition probabilities using the equation (37): OR=p/(1-p) in amyloid positive/p/(1-p) in amyloid negative. This calculation yielded a transition probability of 10% from SCD to MCI and 5% from MCI to mild dementia in amyloid-negative individuals.

##### Dementia disease progression

We analysed transition probabilities between mild, moderate, severe dementia, and institutionalisation using data from SveDem (38). The description of the dataset and analyses were detailed in Section 2 of the supplementary file.

#### Mortality

We used age-specific mortality data for the general population (39) and Hazard Ratios (HRs) for mortality derived from SveDem to establish transition probabilities to death. The analysis included 64,172 observations from a cohort of 37,718 individuals diagnosed with AD in the SveDem dataset. Dementia states were categorised into very mild (MMSE 27–30), mild (MMSE 21–26), moderate (MMSE 10–20), and severe (MMSE 0–9). These states, combined with institutionalisation status, created a total of eight distinct health states for our analysis. To estimate HRs for mortality, we employed a Cox proportional hazard model and the results are presented in Table S4. We implemented Inverse Probability of Censoring Weights to address the potential issue of selective dropout (40). The analysis used very mild dementia as a reference, to obtain HRs of death relative to the general population, we multiplied the HRs for each state by the HR of death for very mild dementia (HR - 1.82) from a population-based cohort study by Andersen et al (41). The final HRs of death for each disease stage are presented in Table S5. Individuals with SCD and MCI were assumed to have mortality rates equivalent to those of the general population. Age-specific mortality data was directly applied to these groups in our model. For individuals with clinical dementia, we integrated the HRs for mortality derived from the analysis with age-specific mortality data for the general population using the formula: 1-exp(-age specific annual death probability*HR of death for disease stage).

#### Sensitivity and specificity of BBM testing

The sensitivity and specificity values for BBM were obtained from a study that compared plasma Aβ40, Aβ42, and tau levels, as measured by Elecsys immunoassays, to the CSF Aβ42/Aβ40 ratio for detecting brain amyloid accumulation (32). In this study, the authors used CSF Aβ42/Aβ40 ratio as a reference test to evaluate the diagnostic accuracy of various combinations of plasma biomarkers. The evaluation was conducted in two cohorts comprising individuals with SCD, MCI, and dementia. For our model, we applied a combination of 89% (80% – 95%) for sensitivity and 69% (54% – 81%) for specificity for BBM testing.

#### Sensitivity and specificity of CSF biomarker testing

The sensitivity and specificity values for CSF biomarkers were derived from a study that examined the concordance of CSF amyloid results with PET imaging (31). In this study, the sensitivity was found to be 91% and the specificity was 89%.

#### Cost Estimations

All cost values were converted to euros (€) (1€ = 1.1 US$, 1 € = 0.087 Swedish kronor (SEK)) and 2022 values using the Consumer Price Indices (CPI) published by Statistics Sweden. The conversion was accomplished through the following formula: Cost in 2022=cost in original year*(2022 CPI/ original year CPI (42)

#### Diagnosis cost

The cost for diagnosis was calculated based on the work by Wimo et al. and was specific to the Swedish healthcare setting (25). This diagnosis cost at PHC included expenses related to consultations, MMSE and laboratory examinations. Additional costs for structural imaging were included for individuals with suspected MCI or dementia, considering that either CT or MRI might be required to rule out other potential causes of their symptoms. The cost of CSF included the cost of consultation at MC plus the cost of CSF investigation assuming that CSF analysis would only be available and prescribed following consultation at MC.

#### Disease-specific cost

The annual cost associated with MCI was adapted from a study conducted by Sköldunger et al (43). This same cost was applied to the SCD state, assuming that SCD shares similar expenses with MCI. Annual costs for mild, moderate, and severe dementia were derived from a recent meta-analysis on the cost of dementia in Europe by Jönsson et al (44). The cost figures were categorized by disease stage (mild, moderate, severe), care setting (community or institutionalization), and European region. For our model, we used values from the Nordic region to represent state-specific costs. To note, separate costs for amyloid-positive and negative patients were not assigned since a study indicated that costs did not differ by amyloid status (45).

#### Cost for disease-modifying treatment

We assumed the annual DMT price to be €5,000, which was obtained by a price threshold analysis at the WTP threshold of €50,000 (see Table S6 in supplementary file). This was because the current announced prices for DMTs (46) are not cost-effective and interfere with the model results.

#### DMT treatment effect and duration

Data from aducanumab and lecanemab clinical trials was used to model DMT effect and duration (3, 4). DMT was prescribed to individuals with MCI due to AD and mild AD dementia. A treatment effect of 27% reduction in disease progression from MCI to mild and mild to moderate AD was applied. Treatment duration is 2 years or until progression to moderate AD whichever comes first. No residual treatment effect was considered. The model assumed a 100% treatment response in persons with abnormal amyloid and 0% treatment response in persons with normal amyloid without considering possible different response rates for AD subtypes.

#### Health utilities

We applied different health utility values for amyloid-negative and amyloid-positive SCD and MCI in our model. These values were sourced from estimates provided by the Swedish BioFINDER study (47). For individuals in different stages of AD, we utilised health utility values derived from a comprehensive systematic review conducted by Landeiro et al (48). The study reported health utilities based on the EQ-5D instrument for MCI, mild, moderate, and severe AD. We applied the caregiver-rated values from this study to our model.

## Results

### Base case analysis

**Figure 3:**
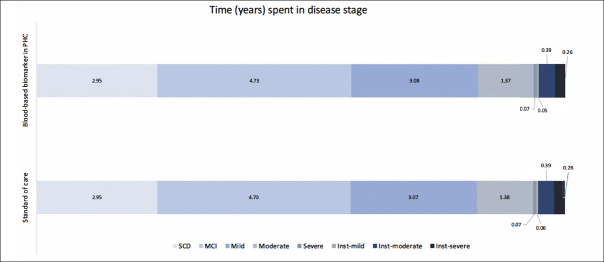
Time (years) spent in each disease stage Abbreviation: SCD, subjective cognitive decline; MCI, mild cognitive impairment; Inst, institutionalization.

Table 2 presents the base case analysis from the decision analytic model. Compared to SOC, using BBM as a decision tool for MC referrals resulted in an 8% increase in referrals and CSF examinations. Consequently, the rate of TP AD diagnoses increased by 10.4%, and TN AD diagnoses increased by 0.4%. When considering downstream decision-making and long-term health outcomes for each diagnostic strategy, the lifetime costs for individuals diagnosed with AD were €249,695 in the SOC and €250,287 in the PHC-BBM pathway, while Quality-Adjusted Life Years (QALYs) were 9.5 and 9.52, respectively. Implementing BBM as a PHC referral decision tool increased costs by €603 and QALYs by 0.01, resulting in an Incremental Cost-Effectiveness Ratio (ICER) of €48,296. When looking at the cost contribution, diagnosis cost contributed to less than 1% of the total cost in both pathways, while DMT cost contributed to the major cost difference between the two pathways. Figure 3 illustrates the time spent in each disease stage. The time living with AD was 12.88 and 12.89 years in SOC and BBM arms, with the differences observed primarily in MCI stage.

**Table 2:** Base case analysis comparing standard of care diagnostic pathway and using blood biomarker as referral decision tool in primary health center

Parameter	Standard of care	Blood biomarker in primary health center	Difference
Intermediate outcomes			
Percentage of refer to memory clinic	24.4%	32.4%	8.0%
Percentage received CSF examination	24.4%	32.4%	8.0%
Percentage of AD TP	14.6%	24.9%	10.4%
Percentage of AD TN	51.9%	52.2%	0.4%
Percentage of AD FP	0.9%	0.6%	−0.4%
Percentage of AD FN	32.6%	22.3%	−10.4%
Cost-effectiveness analysis			
Cost			
Total cost	€ 249,685	€ 250,287	€ 603
Diagnosis cost	€ 1,874	€ 2,122	€ 249
Disease cost	€ 246,341	€ 245,749	−€ 592
DMT cost	€ 1,470	€ 2,416	€ 946
Life years	12.88	12.89	0.01
Quality-Adjusted Life Years (QALYs)	9.50	9.52	0.01
Incremental Cost-Effectiveness Ratio (ICER)			€ 48,296


Abbreviation: TP, true positive; TN, true negative; FP, false positive; FN, false negative.

Table 3 presents the results of an analysis that compares the alternative use of BBM in MC as a triaging tool for CSF examination to SOC. Using BBM in MC to guide CSF examination could reduce the exposure of individuals to invasive CSF procedures by 7.6% and reduce the proportion of TP AD diagnoses by 1.6% but increase TN AD diagnoses by 0.6%. When considering long-term health outcomes in the model, this strategy was associated with €178 lower costs and a slight reduction of 0.002 QALYs compared to SOC. This led to an ICER of €92,253, positioning it in the southwest quadrant of the cost-effectiveness plane, indicating this strategy is inferior to SOC at the WTP threshold.

**Table 3:** Analysis comparing standard of care diagnostic pathway and using blood biomarkers in memory clinic as a triaging test for CSF biomarker examination

Parameter	Standard of care	Blood biomarker in Memory clinic	Difference
Intermediate outcomes			
Percentage of refer to memory clinic	24.40%	24.40%	0
Percentage received CSF examination	24.40%	16.90%	−7.60%
Percentage of AD TP	14.60%	13%	−1.60%
Percentage of AD TN	51.90%	52.50%	0.60%
Percentage of AD FP	0.90%	0.30%	−0.60%
Percentage of AD FN	32.60%	34.30%	1.60%
Cost-effectiveness analysis			
Cost			
Total cost	€ 249,685	€ 249,507	−€ 178
Diagnosis cost	€ 1,874	€ 1,818	−€ 56
Disease cost	€ 246,341	€ 246,433	€ 92
DMT cost	€ 1,470	€ 1,257	−€ 214
Life years	13.700	13.696	−0.004
Quality-Adjusted Life Years (QALYs)	9.504	9.502	−0.002
Incremental Cost-Effectiveness Ratio (ICER)			€ 92,253


Abbreviation: TP, true positive; TN, true negative; FP, false positive; FN, false negative.

### Sensitivity analysis

**Figure 4:**
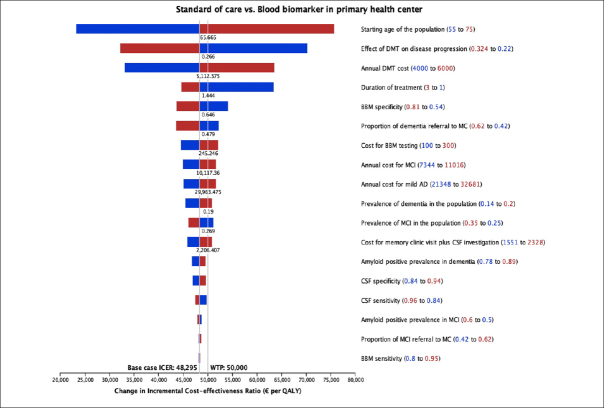
Tornado diagram from deterministic sensitivity analysis

Figure 4 displays the results from the deterministic sensitivity analysis, where individual parameters were varied one at a time. Notably, the starting age of the population, treatment effectiveness, cost and treatment duration of DMT, and BBM specificity showed the most influence on ICER. Factors such as the prevalence of MCI and dementia, the prevalence of amyloid positivity, test sensitivity and specificity, and diagnosis-related costs had less influence (<10%) on the ICER.

**Figure 5:**
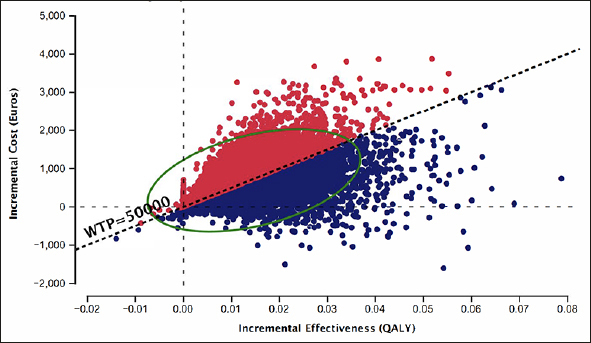
Probabilistic sensitivity analysis from 10,000 iterations, Blood biomarker in primary health center vs. Standard of care

Figure 5 depicts the probabilistic sensitivity analysis from 10,000 iterations. Using BBM in PHC as a diagnostic strategy is more costly and effective than the SOC diagnostic pathway. 44% of the simulated ICERs were above, and 48% fell below the WTP threshold of €50,000.

## Discussion

### Impact on correct diagnosis and cost

The study revealed that implementing BBM as a referral decision tool in PHC increased the proportion of patient referrals and CSF examinations at MC. This finding contrasts with other models, which suggested that using BBM for referrals might reduce patients at MC (22, 23). The primary difference comes from the assumption about patient referrals in the SOC pathway. Previous studies assumed that all symptomatic individuals would be referred to MC for a definitive AD diagnosis. Therefore, adding BBM to the intervention reduced referrals by ruling out symptomatic individuals with a low likelihood of amyloid-positivity. However, this assumption did not align with current clinical diagnostic practices for AD diagnosis. Although referral practices to MC differ by health systems (9, 10, 49), existing literature indicates that not all patients are referred to specialist MC and diagnoses mainly rely on clinical diagnosis in PHC, resulting in low diagnostic accuracy (50). In our model, we adapted it to the Swedish setting where PHC referred only patients who needed a definitive AD diagnosis in SOC practice. Adding BBM in this setting subsequently increases the referrals by ruling in possible amyloid-positive individuals who might otherwise be missed by current SOC practice or be considered AD without the need to refer. To translate this into population level, we assume 10,000 individuals with SCC seeking diagnostic evaluation within one year, implementing BBM in PHC results in an additional 800 patients being referred to MC, requiring lumbar punctures and CSF examinations. This incurs an additional cost of €2.4 million for diagnosis. If we consider diagnostic accuracy, integrating BBM into PHC diagnosis workouts can identify 81% of the prevalent amyloid-positive MCI and mild AD cases within the population, whereas the SOC approach could detect only 47% of these cases. Over half of the prevalent population would remain undiagnosed for abnormal amyloid with the current SOC approach, representing a missed opportunity to initiate effective DMT and incurring €20.6 million in costs due to disease progression within one year that could have been prevented by treatment. See Table S7 and Table S8 of supplementary file for details.

Conversely, using BBM in MC as a triaging tool for CSF examination could reduce exposure to invasive CSF examination but lead to a decrease in correctly diagnosed amyloid-positive cases. This was to be expected as only individuals who tested positive for BBM would undergo CSF examination. This approach saves around €0.6 million for initial diagnosis but incurs €3.4 million in costs associated with disease progression that could have been prevented by treatment (see Table S9 and Table S10 in supplementary file). This is compared to SOC diagnosis, which is inadequate in the presence of an effective DMT. If comparing this scenario with using BBM in PHC, the cost of preventable disease progression due to misdiagnosis would be €24 million. Therefore, using BBM in PHC might increase initial diagnostic evaluation costs but can prevent the high costs associated with misdiagnosis and disease progression, whereas using BBM in MC could reduce the initial evaluation cost but incur high costs associated with disease progression.

### Effect of DMT on cost-effectiveness of BBM

The use of BBM in PHC as a referral decision tool in our model showed an ICER just below the WTP threshold. That was because we applied a cost-effective DMT price of €5,000 estimated from price threshold analysis. Sensitivity analysis from the model showed that a little change in DMT cost could greatly influence the ICER. When applying the current DMT set price of €24,910, the resulting ICER is significantly higher than the WTP threshold (Table S11 in supplementary file), masking the potential benefits of correct diagnosis. This current set price of DMT has been suggested by European Alzheimer's Disease Consortium Executive Committee as unsustainable relative to the benefits gained (51). Apart from the cost, the treatment effect and duration also greatly influence the ICER. In our model, we assumed a treatment duration of 2 years and did not consider residual treatment effect (i.e., the effect did not persist after 2 years) using data from the 18-month clinical trial. In a sensitivity analysis, we assumed the treatment effect to persist for 3 years, which resulted in a decreased ICER. Therefore, a cost-effective treatment should be in place for BBM to be cost-effective.

### Sensitivity and Specificity of BBM

The sensitivity analysis indicated that higher BBM specificity reduces the ICER. This was because higher BBM specificity reduces referral to MC, reducing FP diagnosis, thus avoiding unnecessarily high costs for prescribing DMT to FP individuals (refer to Table S12 in supplementary file). Higher sensitivity had almost no impact on the ICER. These sensitivity results are limited because the conditional performance of tests was not considered (i.e., performance of CSF conditional on BBM being positive or negative). In general, BBM (and CSF) are based on a cut-off that drives the combination of sensitivity and specificity (typically seen in a ROC-curve), where increased sensitivity comes at the cost of specificity and vice versa (52). Wimo suggested a highly sensitive screening test at PHC followed by a highly specific confirmatory test at MC (53). That approach will ensure that individuals who are currently not referred to MC and thus miss the opportunity for treatment benefits are referred, albeit at the cost of potentially overburdening MC with more referrals. Furthermore, the CSF test in MC will make sure a very expensive treatment will not be unnecessarily provided to those who will not benefit. The optimisation is to get more people who are now missed by PHC due to low referrals than those who missed out by being stricter in the alternative pathway of using CSF in MC.

Important factors that can potentially influence the sensitivity and specificity of BBM and need to be considered in the model are patient clinical characteristics and APOE ε4 carrier status. AD is a chronic disease that usually exists with other comorbidities. The starting population in our model is people with SCC seeking care at PHC. The population is younger and might have other comorbidities interfering with the BBM results. Studies have indicated that BBM levels tend to increase with age and are elevated in individuals with APOE ε4 allele, chronic kidney disease, hypertension, stroke, myocardial infarction (54) and higher body mass index (55). These studies have also proposed that such variation could potentially be mitigated by defining specific cut-off values for different age groups and specific clinical conditions or by employing 2 cut-offs rather than a single cut-off in interpreting BBM results. A recently published paper has demonstrated that the performance of plasma %p-tau 217 is improved with two cut-off approaches that categorise individuals into positive, negative or indeterminate amyloid groups (56).

Our model is a simplification of the real-world clinical setting. We applied simple dichotomised values, i.e., test sensitivity and specificity, based on a single cut-off in the model (32). With this approach, the test results are categorised into true positive and false positive, true negative or false negative. The group of people who might have indeterminate BBM results due to clinical characteristics fall into false positive and false negative diagnoses. Their costs and outcomes were reflected through the subsequent clinical decisions and disease progression in the model. If a 2-cut-offs approach is applied in the simulation that defines patients into high, intermediate, and low probabilities of having AD, as suggested by Hansson et al. (20), the cost-effectiveness estimates from the model might be more accurate. However, the current understanding of BBM for AD requires further validation through prospective clinical studies and establishment of cut-off values that are simple for clinical interpretation and decision-making. Future research efforts aimed at defining cut-off values for BBM might hold promise for facilitating clinical decision-making and enhancing the precision of economic models.

### Strengths and limitations

This study represents the first attempt to assess the cost-utility of implementing BBM in clinical settings where DMT is available. It addresses an important gap in the literature, offering valuable insights into the potential benefits and costs of incorporating BBM into diagnostic pathways. The model's design is grounded in clinical guidelines and utilises registry data, enhancing its credibility and relevance to real-world clinical practice. However, due to the heterogeneity in AD referral and diagnosis practice, the findings can only reflect the Swedish setting or health system alike.

Moreover, economic models are inherently limited by data availability and their methodological assumptions. Firstly, the model exclusively considers AD-type dementia, which might not fully reflect the complexity of real-world clinical settings where patients with memory complaints may have various comorbidities and neurodegenerative diseases. This assumption might underestimate the impact of delayed diagnosis for other conditions and the effect of certain types of comorbidity on BBM results. Secondly, the model treats diagnosis as a one-time process, with individuals receiving costly DMT following a FP diagnosis and those with FN results missing out on treatment. This simplification may not fully capture the complexities of real-world clinical practice, where individuals with FN results may return for further assessments if their symptoms persist or worsen. Thirdly, there are methodological uncertainties related to the DMT model. The model simplifies the disease-modifying nature of DMT by representing it as a reduction in disease progression without considering residual treatment effects or considering direct effects on institutionalisation and mortality. Fourthly, the model assumes that all biomarker-confirmed AD patients will respond to treatment. Another model has assessed the cost-effectiveness of DMT assuming different hypothetical treatment responses by AD subtypes (57). Most importantly, in the intervention arm, we used BBM positivity as the sole criterion for referral without considering potential correlations between PHC physicians' assessments and amyloid positivity. While PHC does not perform amyloid tests, information used in current practice to set an AD diagnosis likely correlates with amyloid positivity. For instance, younger individuals with cognitive impairment have a higher risk of amyloid positivity than older individuals without cognitive impairment, and evidence has shown that information from medical history is associated with disease progression (52, 58). However, since the accuracy of PHC physicians in predicting amyloid positivity using current tests is presumed to be low, relying solely on BBM test results to model referrals seems a plausible scenario. Similarly, the model did not account for test dependency between BBM and CSF biomarkers. For instance, CSF sensitivity was the same for all patients and did not vary based on BBM-positive results. These limitations were attributed to the unavailability of data. Ideally, data on physician's judgement on the likelihood of amyloid positivity, BBM amyloid positivity, CSF amyloid positivity and a reference standard (e.g., amyloid PET) are all available cross-sectionally within the same individual. The sensitivity and specificity data applied in the model were sourced from different references, with BBM values referencing CSF and CSF values referencing PET and no empirical evidence supporting physician judgement. Consequently, we were unable to compare the sensitivity and specificity of a test sequence. For example, if the sensitivity and specificity for a sequence involving physician judgment + BBM + CSF are identical to those for clinician judgment + BBM, the addition of a CSF test following BBM could be considered redundant and inefficient. Nonetheless, the primary goal of the model is to provide insights into the consequences of integrating BBM into the current AD diagnostic workflow, and in this context, the model effectively fulfils its intended purpose. Future research that provides this essential information would enhance our understanding of the diagnostic sequence.

## Conclusion

In conclusion, the study demonstrates the potential benefits and costs of integrating BBM into clinical settings. In a setting where PHC referral goals are for AD diagnosis and DMT initiation, implementing BBM in PHC can effectively reduce false positive and false negative amyloid abnormality and avoid costs associated with disease progression, whereas using BBM in MC as a triaging test for CSF examination may reduce the initial evaluation cost and patient flow to MC but result in high costs associated with care costs related to unprevented disease progression. Understanding current referral practice and diagnostic pathways is important to model the impact of BBM in AD diagnosis.

## Electronic supplementary material

Optimising Alzheimer's disease diagnosis and treatment: assessing cost-utility of integrating blood biomarkers in clinical practice for disease-modifying treatment

*Conflicts of interest disclosure:* Sandar Aye has no competing interest to declare. Ron Handels reports the following outside the study: Funding (paid to department) from (private-)public frameworks JPND, IMI, H2020 (EU), ZonMw (NL), partly via affiliation at Karolinska Institutet; consulting fees (paid to department) from Lilly Nederland B.V. (advisory; 2022), institute for Medical Technology Assessment (advisory; 2021; content initiated by Biogen), Biogen Netherlands BV (advisory; 2021), Biogen MA Inc. (advisory; 2020), Eisai Inc. (advisory; 2019); Member of ISPOR open-source modeling Special Interest Group (co-chair; 2022-now; unpaid), member of IPECAD modeling group (steering group; 2017-now; unpaid). Bengt Winblad has received license fees for the Resource Utilization in Dementia (RUD) instrument. Consulting fees for SAB meetings with AlzeCure, Alzinova, NovoNordisk and Axon Neuroscience. Linus Jönsson has received consulting fees from H. Lundbeck A/S and license fees for the Resource Utilization in Dementia (RUD) instrument.

*Ethical approval statement:* No ethics approval was required for this study because it uses existing evidence and registry data. However, we have obtained permission for related data collection from the Stockholm Regional Ethics Review Board (DNR 2023-02485-01).
