# Supplementary material for: Optimising Alzheimer’s Disease Diagnosis and Treatment: Assessing Cost-Utility of Integrating Blood Biomarkers in Clinical Practice for Disease-Modifying Treatment
Source: J Prev Alzheimers Dis. 2024 Apr 10;11(4):928–42. doi: 10.14283/jpad.2024.67 (PMC11266371; doi:10.14283/jpad.2024.67)
Supplement: Supplementary file 1 [file 42414_2024_339_MOESM1_ESM.pdf]

Supplementary file for the manuscript titled " Optimising Alzheimer's disease diagnosis and treatment: assessing cost-utility of integrating blood biomarkers in clinical practice for disease-modifying treatment"

Authors and affiliations:

Sandar Aye[1], Ron Handels[1,2], Bengt Winblad[1,3], Linus Jönsson[1]

[1] Division of Neurogeriatrics, Department of Neurobiology, Care Sciences and Society, Karolinska Institutet, BioClinicum J9:20, Akademiska stråket 171 64 Solna, Sweden

[2] Department of Psychiatry and Neuropsychology; Maastricht University; Alzheimer Centre Limburg; Faculty of Health, Medicine and Life Sciences; School for Mental Health and Neuroscience; 6200 MD, Maastricht, The Netherlands

[3] Theme Inflammation and Aging, Karolinska University Hospital, Blickagången 6, 14157, Huddinge, Sweden

Corresponding authors: Sandar Aye

Email: sandar.aye@ki.se

Phone: +46 704347761

Address: Karolinska Institutet, BioClinicum J9:20, Akademiska stråket 171 64 Solna, Sweden

Section 1. Diagnosis of Alzheimer’s disease in Sweden

A diagnosis of Alzheimer’s Disease (AD) in Sweden begins with basic dementia assessments in PHC, including symptom assessments, cognitive tests (such as the Mini-Mental State Examination (MMSE)), and laboratory examinations to rule out other diseases (1). If suspicion of cognitive impairment persists, structural imaging (CT brain) and assessments of functional abilities are conducted. Clinical diagnoses are based on ICD-10 criteria (2). Individuals diagnosed with MCI or AD dementia are followed up in PHC. Those with inconclusive basic dementia assessments, younger individuals, or those with unclear causes of MCI or dementia are referred to MC for extended AD assessments. These include in-depth neuropsychological evaluations, brain MRI, cerebrospinal fluid (CSF) biomarkers, and positron emission tomography (PET) (1). According to a study estimating resource constraints in dementia diagnosis, the clinical specialists involved in diagnosing patients with dementia in MC include geriatricians, psychiatrists, and neurologists (3).

Table S1. Consequences of final diagnosis

True cognitive state	True underlying Amyloid status	Final diagnosis	Decision for disease-modifying treatment	Effect on progression	Consequences on cost
SCD	Negative	SCD - unspecified	Don't treat	No progression	
SCD	Positive	SCD - unspecified	Don't treat	Fast progression	
MCI	Negative	AD FP	Treat	No progression	+ treatment cost
		AD TN	Don't treat	No progression	
MCI	Positive	AD TP	Treat	Slowed progression	+ treatment cost
		AD FN	Don't treat	Fast progression	
Mild dementia	Negative	AD FP	Treat	No progression	+ treatment cost
		AD TN	Don't treat	No progression	
Mild dementia	Positive	AD TP	Treat	Slowed progression	+ treatment cost
		AD FN	Don't treat	Fast progression	

Abbrebiations: SCD, subjective cognitive decline; MCI, mild cognitive impairment; AD, Alzheimer's disease; FP, false positive; TP, true positive; FN, false negative; TN, true negative.

Section 2. Transition Probabilities for Mild, Moderate, Severe, and Institutionalization

We identified 153,014 observations from 81,844 dementia patients from 2007 to December 2018. >95% of the study participants did not have a recorded institutionalisation status. We regarded the status as not institutionalised since >85% of all followed-up observations were not institutionalised. Observations with missing baseline MMSE and incorrect follow-up date were removed. Missing MMSE information was imputed with previous MMSE if the interval of follow-up is less than or equal to one year, observations still having missing MMSE after the imputation were removed. After excluding observations with missing baseline or any recorded MMSE, follow-up duration less than 4 months (as have been done in the previous study) (4) or longer than 12 months, 49,172 annualised transitions were included in the analysis. Dementia state was defined by using MMSE cutoffs at MMSE 21-30, MMSE 10-20 and MMSE 0-9 for mild, moderate and severe dementia. Institutionalisation was defined as living in special accommodations or permanent special housing adapted for people with dementia. The age-specific transition probabilities to each health state were estimated by multivariate ordered probit regression model and shown in Table S2. Institutionalisation was modelled as a distinct health state. Therefore, transition probabilities for 6 health states (mild, moderate, severe, institutionalised mild, institutionalised moderate and institutionalised severe) were estimated from the model. Backward transitions from institutionalisation to community are not allowed, and these were regarded as staying in the same health state. Table S3 displays the transition probabilities at age 65, which we applied in our model to represent the model population.

Table S2. Result of ordered probit regression using SveDem data

Variable		Coefficient (Standard error)	P value
Clinical stage			
	Mild	Reference	
	Mild institutionalised	3.15 (0.04)	<0.01
	Moderate	1.79 (0.01)	<0.01
	Moderate institutionalised	4.33 (0.04)	<0.01
	Severe	2.98 (0.03)	<0.01
	Severe institutinalised	7.28 (0.19)	<0.01
Age at diagnosis		0 (0)	<0.01
Female		0.06 (0.01)	<0.01
Cut			
	Mild	Moderate	1 (0.06)	<0.01
	Moderate	Severe	2.98 (0.06)	<0.01
	Severe	Mild institutionalised	3.23 (0.06)	<0.01
	Mild institutionalised	Moderate institutionalised	3.57 (0.06)	<0.01
	Moderate institutionalised	Severe institutionalised	5.59 (0.07)	<0.01

Table S3. Annual transition probability based on ordered probit regression coefficients

		To					
From	States	Mild	Mode rate	Sever e	Mild institutio nalized	Moderate institution alized	Severe institutinali zed
	Mild	0.803	0.194		0.001		
	Moderate	0.177	0.675	0.051		0.097	
	Severe		0.426	0.117			0.457
	Mild institutionalized				0.608	0.392	
	Moderate institutionalized				0.076	0.791	0.133
	Severe institutinalized					0.033	0.967

NOTE. transition to one more state forward or backward is combined as one, for example, transition from mild to severe was combined as transition to moderate and so on.

Table S4. Survival analysis using SveDem data, 64,178 observations from 37,718 patients, 15,422 died, Cox proportional hazard model adjusted for age and sex, corrected for selective drop out

Variable		Hazard ratio	95% confidence interval	P value
Clinical stage				
	Very mild	Reference		
	MiId	1.27	1.19 - 1.35	<0.001
	Moderate	1.84	1.72 - 1.97	<0.001
	Severe	2.94	2.63 - 3.28	<0.001
	Very mild - institutionalized	1.1	0.69 - 1.76	0.695
	Mild - institutionalized	2.01	1.75 - 2.31	<0.001
	Moderate - institutionalized	2.98	2.7 - 3.3	<0.001
	Severe - institutionalized	3.44	2.76 - 4.29	<0.001
Age at diagnosis		1.08	1.08 - 1.08	<0.001
Female		0.67	0.64 - 0.69	<0.001

Table S5. Hazard ratios (HR) of death for each disease stage after integrating HR of death for very mild dementia

Clinical stage	HR of death
SCD	1
MCI	1
Mild	2.31
Moderate	3.35
Severe	5.35
Mild - institutionalised	3.66
Moderate - institutionalised	5.42
Severe - institutionalised	6.26

Table S6. DMT price threshold analysis, treat all MCI and mild dementia with true underlying amyloid

**WTP**	**Price threshold to be cost-effective at the set WTP**
€ 50,000	€ 5,112
€ 80,000	€ 7,090
€ 100,000	€ 8,409

Table S7. Calculation for cost incurred by diagnostic evaluation assuming a 10,000 population seeking diagnostic evaluation in each arm

**Strategy**	Standard of care	Blood biomarker in primary health center	Difference
Number of memory clinic referral	2440	3240	800
Number received CSF examination	2440	3240	800
Number of AD TP diagnosis	1460	2490	1030
Number of AD TN diagnosis	5190	5220	30
Number of AD FP diagnosis	90	60	-30
Number of AD FN diagnosis	3260	2230	-1030
Cost for diagnosis evaluation	€ 18,735,260	€ 21,223,846	€ 2,488,586

Table S8. Calculation for cost incurred due to disease progression assuming a 10,000 population seeking diagnostic evaluation in each arm

	Number of amyloid positive in the population	SOC detection	BBM - PHC detection	Difference in detection	Cost for progression per patient	Cost for progression
MCI	1650	780	1340	560	€ 13,104	€ 7,337,998
Dementia	1411	680	1160	480	€ 27,808	€ 13,347,679
MCI & dementia	3061	1460 (47%)	2500 (81%)			€ 20,685,677

Table S9. Calculation for cost incurred by diagnostic evaluation assuming a 10,000 population seeking diagnostic evaluation in each arm

**Strategy**	Standard of care	Blood biomarker in memory clinic	Difference
Number of memory clinic referral	2400	2400	0
Number received CSF examination	2400	1680	-720
Number of AD TP diagnosis	1460	1300	-160
Number of AD TN diagnosis	90	30	-60
Number of AD FP diagnosis	5190	5250	60
Number of AD FN diagnosis	3260	3420	160
Cost for diagnosis evaluation	€ 18,735,260	€ 18,176,455	-€ 558,805

Table S10. Calculation for cost incurred due to disease progression assuming a 10,000 population seeking diagnostic evaluation in each arm

	Number of amyloid positive in the population	SOC detection	BBB-MC detection	Difference in detection	Cost for progression per patient	Cost for progression
MCI	1650	780	690	-90	€ 13,104	-€ 1,179,321
Dementia	1411	680	600	-80	€ 27,808	-€ 2,224,613
MCI & dementia	3061	1460 (47%)	1290 (42%)			-€ 3,403,934

Table S11. Cost-effectiveness analysis using current set price for DMT (Annual DMT cost = €24910) (5)

**Strategy**	**QALYs**	**Total cost**	**ICER**
Standard of care	9.50	€ 255,539	
Blood biomarker in PHC	9.52	€ 259,909	€ 350,248

Table S12. Change in true positive diagnosis rate based on different BBM sensitivity and specificity when using BBM at primary health center as a referral decision tool

BBM sensitivity and specificity	% Refer to MC	% true positivie (TP)	% false positive (FP)	% true negative (TN)	% false negative (FN)
Base case (sensitivity = 0.89, specificity = 0.69)	32.4	24.9	0.6	52.2	22.3
Higher sensitivity = 0.95, same specificity = 0.69	34.3	26.6	0.6	52.2	20.6
Higher BBM specificity = 0.81, same sensitivity = 0.89	30.5	24.9	0.3	52.5	22.3

References

1. National clinical knowledge support. National clinical knowledge support. 2023 [cited 2023 Aug 25]. Cognitive impairment in suspected dementia - National clinical knowledge support. Available from: https://www.nationelltklinisktkunskapsstod.se/kunskapsstod/vardforlopp/?uuid=c38fa4a0-a3ec-4b15-8aec-beca2fefe69b
2. Socialstyrelsen. Nationella riktlinjer 2017. Vård och omsorg vid demenssjukdom - stöd för styrning och ledning. [National guidelines for care in cases of dementia]. 2017.
3. Mattke S, Gustavsson A, Jacobs L, Kern S, Palmqvist S, Eriksdotter M, et al. Estimates of Current Capacity for Diagnosing Alzheimer's Disease in Sweden and the Need to Expand Specialist Numbers. J Prev Alz Dis. 2023;
4. Wimo A, Handels R, Winblad B, Black CM, Johansson G, Salomonsson S. Quantifying and Describing the Natural History and Costs of Alzheimer's Disease and Effects of Hypothetical Interventions. J Alzheimers Dis. 2020;75(3):891–902.
5. Eisai. Eisai. U.S. 2023 [cited 2023 Jun 28]. EISAI'S APPROACH TO U.S. PRICING FOR LEQEMBI™ (LECANEMAB), A TREATMENT FOR EARLY ALZHEIMER'S DISEASE, SETS FORTH OUR CONCEPT OF "SOCIETAL VALUE OF MEDICINE" IN RELATION TO "PRICE OF MEDICINE." Available from: https://us.eisai.com/media-center
